# [^18^F]-THK5351 PET Correlates with Topology and Symptom Severity in Progressive Supranuclear Palsy

**DOI:** 10.3389/fnagi.2017.00440

**Published:** 2018-01-17

**Authors:** Matthias Brendel, Sonja Schönecker, Günter Höglinger, Simon Lindner, Joachim Havla, Janusch Blautzik, Julia Sauerbeck, Guido Rohrer, Christian Zach, Franziska Vettermann, Anthony E. Lang, Lawrence Golbe, Georg Nübling, Peter Bartenstein, Katsutoshi Furukawa, Aiko Ishiki, Kai Bötzel, Adrian Danek, Nobuyuki Okamura, Johannes Levin, Axel Rominger

**Affiliations:** ^1^Department of Nuclear Medicine, Ludwig-Maximilians-Universität München, Munich, Germany; ^2^Department of Neurology, Ludwig-Maximilians-Universität München, Munich, Germany; ^3^German Center for Neurodegenerative Diseases (DZNE), Munich, Germany; ^4^Department of Neurology, Technical University of Munich, Munich, Germany; ^5^Institute of Clinical Neuroimmunology, Biomedical Center and University Hospital, Ludwig-Maximilians-Universität München, Munich, Germany; ^6^Morton and Gloria Shulman Movement Disorders Clinic and the Edmond J. Safra Program in Parkinson's Disease, and Toronto Western Hospital, University of Toronto, Toronto, ON, Canada; ^7^Department of Neurology, Rutgers Robert Wood Johnson Medical School, New Brunswick, NJ, United States; ^8^Division of Community Medicine, Tohoku Medical and Pharmaceutical University, Sendai, Japan; ^9^Department of Geriatrics and Gerontology, Institute of Development, Aging and Cancer, Tohoku University, Sendai, Japan; ^10^Division of Pharmacology, Faculty of Medicine, Tohoku Medical and Pharmaceutical University, Sendai, Japan

**Keywords:** PET, [^18^F]-THK5351, progressive supranuclear palsy, PSPRS, disease severity

## Abstract

Progressive supranuclear palsy (PSP) is a neurodegenerative movement disorder characterized by deposition of fibrillar aggregates of 4R tau-protein in neurons and glial cells of the brain. These deposits are a key neuropathological finding, allowing a diagnosis of “definite PSP,” which is usually established post mortem. To date criteria for clinical diagnosis of PSP *in vivo* do not include biomarkers of tau pathology. For intervention trials, it is increasingly important to (i) establish biomarkers for an early diagnosis and (ii) to develop biomarkers that correlate with disease progression of PSP. [^18^F]-THK5351 is a novel PET-ligand that may afford *in vivo* visualization and quantification of tau-related alterations. We investigated binding characteristics of [^18^F]-THK5351 in patients with clinically diagnosed PSP and correlate tracer uptake with clinical findings. Eleven patients (68.4 ± 7.4 year; *N* = 6 female) with probable PSP according to current clinical criteria and nine healthy controls (71.7 ± 7.2 year; *N* = 4 female) underwent [^18^F]-THK5351 PET scanning. Voxel-wise statistical parametric comparison and volume-of-interest based quantification of standardized-uptake-values (SUV) were conducted using the cerebellar cortex as reference region. We correlated disease severity as measured with the help of the PSP Rating Scale (PSPRS) as well as several other clinical parameters with the individual PET findings. By voxel-wise mapping of [^18^F]-THK5351 uptake in the patient group we delineated typical distribution patterns that fit to known tau topology for PSP post mortem. Quantitative analysis indicated the strongest discrimination between PSP patients and healthy controls based on tracer uptake in the midbrain (+35%; *p* = 3.01E-7; Cohen's d: 4.0), followed by the globus pallidus, frontal cortex, and medulla oblongata. Midbrain [^18^F]-THK5351 uptake correlated well with clinical severity as measured by PSPRS (*R* = 0.66; *p* = 0.026). OCT and MRI delineated PSP patients from healthy controls by use of established discrimination thresholds but only OCT did as well correlate with clinical severity (*R* = 0.79; *p* = 0.024). Regional [^18^F]-THK5351 binding patterns correlated well with the established post mortem distribution of lesions in PSP and with clinical severity. The contribution of possible MAO-B binding to the [^18^F]-THK5351 signal needs to be further evaluated, but nevertheless [^18^F]-THK5351 PET may still serve as valuable biomarker for diagnosis of PSP.

## Introduction

Progressive supranuclear palsy (PSP) is a neurodegenerative movement disorder from the group of atypical parkinsonism manifesting with prominent hypokinesia, oculo-motor and balance disturbances, and with behavioral changes (Stamelou et al., 2010). Several clinical phenotypes of PSP have been described. Patients with PSP-Richardson's Syndrome (PSP-RS) are considered to show an especially good clinic-pathological correlation (Respondek and Hoglinger, 2016). While the initial clinical presentation can resemble Parkinson's disease, PSP has a more malignant course, usually leading to death within a decade after onset. Its pathophysiology is characterized by deposition of fibrillar aggregates of 4-repeat tau protein in neurons and glial cells in affected brain areas, i.e., basal ganglia, brainstem, and cerebral cortex (Dickson et al., 2010). The presence of these deposits is a key finding and leads to the neuropathological diagnosis of “definite PSP,” usually established *post mortem*. Current clinical criteria for diagnosis of possible or probable PSP in living patients do not consider tau pathology (Litvan et al., 1996). For future intervention trials, it is becoming increasingly important to establish biomarkers that allow a clear-cut *ante mortem* diagnosis of the underlying molecular pathology. Furthermore, molecular markers are needed that can serve as non-invasive markers of target engagement and disease progression.

Molecular imaging of tau deposits by positron emission tomography (PET) was mainly focused on Alzheimer's disease, the most common tauopathy. The repertoire of PET imaging agents that target tau includes a carbon-11 labeled ligand (Maruyama et al., 2013), and various [^18^F]-fluorinated radiotracers, which present logistic advantages due to their longer physical half-life (Okamura et al., 2013; Xia et al., 2013). To date, the most promising [^18^F]-labeled tau ligands are the pyrdioleindole [^18^F]-AV1451 (T807) and the arylquinoline [^18^F]-THK5351 (Harada et al., 2016), both of which have already been investigated in Alzheimer's disease.

Recent data suggest that by PET tau depositions can also be discerned in atypical parkinsonian disorders (Coakeley and Strafella, 2015), and in PSP in particular (Chiotis et al., 2016; Hammes et al., 2016). Thus, systematic studies of PET imaging should enable a better understanding of the roles played by tau in the progression of atypical Parkinsonism (Villemagne and Okamura, 2016). In Alzheimer's disease, the progression and spatial distribution of tau deposits, as known from neuropathology, were recapitulated in PET studies using [^18^F]-AV1451 (Schwarz et al., 2016). With the [^18^F]-FDDNP tau ligand, proof of principle was obtained that PSP-specific tau distribution patterns can be captured by PET (Kepe et al., 2013). *In vivo* binding of this ligand in PSP brains, however, showed no association with disease severity, which presumably is due to its unfavorable binding affinity (Kuntner et al., 2009) and its lack of specificity for tau aggregates (Tolboom et al., 2010).

Distinct quaternary structures of fibrillary tau in the various tauopathies must be considered if radioligands are evaluated with respect to the particular pathology targeted (Cairns et al., 2007). Alternative splicing leads to 4-repeat tau in PSP as opposed to 3-repeat tau in Pick's disease and balanced 3-/4-repeat tau in Alzheimer's disease (Murray et al., 2014). For example, the tau aggregates of PSP are composed of straight filaments (Chin and Goldman, 1996) to which the PET ligands available bind with different affinities (Harada et al., 2016). Another issue is primary age-related tau depositions, which are not considered pathologic and are typically found in the medial temporal lobe in elderly subjects (Schöll et al., 2016). Therefore, age-matched control data are indispensable for the evaluation of tau-PET in patients (Herholz, 2016). Importantly, very recent findings indicated an off-target binding to monoamine oxidase B (MAO-B), which emphasizes careful interpretation of *in vivo* results, especially when alterations of MAO-B must be assumed (Ng et al., 2017).

The primary aim of this study was to characterize [^18^F]-THK5351 binding in a group of patients with clinically diagnosed PSP-RS with a broad range of severities, in comparison to a group of age-matched neurologically healthy control subjects. Our secondary aim was to correlate individual tracer uptake and clinical findings on the PSP rating scale (PSPRS). Finally, we sought to compare PET results with multimodal assessment by optical coherence tomography (OCT) and magnetic resonance imaging (MRI).

## Materials and methods

### Clinical evaluation

We enrolled 11 patients from the outpatient clinic for neurodegenerative diseases at the Departments of Neurology, Ludwig-Maximilians-Universität München and Technical University of Munich, Munich, Germany with a diagnosis of clinically probable PSP-RS according to current diagnostic criteria (Litvan et al., 1996). Additionally, we enrolled a single case with suspected PSP-progressive non-fluent aphasia (PSP-PNFA) into the study. The control group consisted of nine healthy and age-matched control subjects investigated at Tohoku University School of Medicine in Sendai, Japan. Disease severity was measured with the PSP Rating Scale (PSPRS; score range 0–100, with higher numbers indicating higher severity) (Golbe and Ohman-Strickland, 2007). Furthermore, functional independence was measured using the Schwab and England Activities of Daily Living scale (SEADL) and disease duration was recorded. The cognitive state was assessed by the Mini-Mental State Examination (MMSE). Written informed consent was obtained by German and Japanese participants in accordance with the Declaration of Helsinki. Retrospective analysis of data had been approved by both local ethics committees (Medical Faculty, Ludwig-Maximilians-Universität München, Munich / Tohoku University).

### Imaging protocols

#### Radiosynthesis

Automated production of [^18^F]-THK5351 was performed on a Raytest® SynChrom R&D single reactor synthesizer. The radiochemical yield was 12 ± 4% (not decay-corrected, *n* = 10) and radiochemical purity >99% at the end of the 84 min synthesis. A detailed description of the radiosynthesis is provided in the Supplementary Material.

#### Image acquisition and reconstruction

Images in the patients were acquired using a GE Discovery 690 PET/CT scanner. A prior low-dose CT scan was performed for attenuation correction. Dynamic 3-dimensional emission recordings were acquired during an interval of 30–70 min after intravenous injection of 183 ± 4 MBq [^18^F]-THK5351. PET data were reconstructed iteratively and binned into 5 min frames for visual assessment of possible head movement. The dynamic data was evaluated to compare the previously-established 40–60 min p.i. window (Lockhart et al., 2016) for PSP target regions against alternative 30–50 and 50–70 min time windows.

Imaging in the control subjects was performed on an Eminence STARGATE PET scanner with dynamic 3-dimensional emission recordings during 0–90 min, initiated upon administration of 182 ± 3 MBq [^18^F]-THK5351; 30–50, 40–60, and 50–70 min p.i. frames were iteratively reconstructed. In order to adjust different resolution properties of the two PET-scanners we aimed to perform harmonization after comparison of 3-D Hoffmann brain phantom scans (Joshi et al., 2009). The phantom analysis revealed that a final resolution of 8.0 mm (x/y-axis) and 9.0 mm (z-axis) was achieved for both scanners using the reconstruction parameters. Thus, no further adaption was deemed necessary.

Anatomical MRI in patients was performed using a 3.0 Tesla Magnetom (Signa HDxt, General Electric, Chicago, USA) with a 8-element head coil. A sagittal 3D fast spoiled gradient echo sequence (FSPGR) was acquired with the following imaging parameters: field of view, 240 × 240 mm; spatial resolution, 1 × 1 × 1 mm; time of repetition, 6.616 ms; time of echo, 3.150 ms; flip angle, 15; number of slices, 176.

Anatomical MRI in control subjects were performed using a Signa 1.5-Tesla machine (General Electric). In T1-weighted MR acquisition, a three-dimensional volumetric acquisition of a T1-weighted gradient echo sequence produced a gapless series of thin axial sections using a vascular TOF SPGR sequence (echo time/ repetition time, 2.4/50 ms; flip angle, 45°; acquisition matrix, 256 × 256; 1 excitation; field of view, 22 cm; slice thickness, 2.0 mm).

#### Coregistration and postprocessing

The PNEURO data processing pipeline of PMOD Version 3.5 (PMOD Technologies Ltd., Zurich, Switzerland) was used for spatial normalization of all [^18^F]-THK5351 images to the Montreal Neurological Institute (MNI) space. We first performed co-registration of the individual MRI to a T1w template (Brendel et al., 2015). Next, the 40–60 min PET-images for all cases with available MRI (*N* = 18) were scaled by global mean intensity, and averaged for generation of a mixed PSP/healthy control [^18^F]-THK5351 template in the MNI space. Subsequently, all (*N* = 21) individual PET images were spatially normalized to the [^18^F]-THK5351 template using the PMOD FUSION tool (equal modality; nonlinear warping; 16 iterations; frequency cutoff 3; regularization 1.0; no thresholding; 8 mm transient input smoothing). All co-registered images showed excellent spatial agreement with visual inspection. The final transformation matrices were applied to dynamic data and the other frame intervals for congruent spatial normalization. Given that only sporadic cerebellar tau depositions are reported in PSP when ataxia is absent clinically (Kanazawa et al., 2009), a uniform volume consisting of the cerebellar gray matter (114 cm^3^), excluding the dentate nuclei, was used as the reference region for image intensity normalization.

#### Voxel-wise analyses

Statistical parametric mapping (SPM) was performed using SPM8 implemented in Matlab 7.12.0 (R2011a). PSP-RS patient (*N* = 11) and healthy control (*N* = 9) groups were compared by a voxel-wise two-tailed student's *t*-test after 6 mm Gaussian smoothing. The contrast was performed after masking of extra-cerebral structures, including caudo-dorsal segments of the cerebellum (due to their propensity for edge artifacts), and with exclusion of clusters of less than 20 voxels. Voxels surviving *p* < 0.05 after correction for multiple comparisons (false discovery rate, FDR) were considered significant. Z-score maps for individual patient/control contrasts were generated using PNEURO after having first calculated a normative database (mean/standard deviation, SD), and upon 6 mm Gaussian smoothing. Maps showing Z-scores > 3 were visually displayed using an image overlay tool implemented in Matlab.

#### Semiquantitative PET analyses

For semiquantitative analyses, volumes-of-interest (VOIs) were defined in T-score maps deriving from SPM analyses superimposed on the individual MRIs for anatomical validation. The 3-dimensional assignment of “hot” voxel clusters to VOIs was performed with a T-score threshold of >3.56 (≈SPM *p* < 0.05, FDR). Predefined Hammers atlas VOIs (Hammers et al., 2003) were additionally applied for brainstem, midbrain (extracted from brainstem), and bilateral precentral gyri, superior and inferior frontal gyri, putamen, globus pallidus, and substantia nigra. Mean standard-uptake-value ratios relative to cerebellum (SUVR_CBL_) were calculated for each VOI in all subjects.

### Multimodal analyses

#### Optical coherence tomography and intraretinal layer segmentation

OCT examination was performed using a SD-OCT aparatus (Spectralis, Heidelberg Engineering, Heidelberg, Germany). The OCT protocol included a peripapillary ring scan for measuring peripapillary retinal nerve fiber layer (pRNFL) (12°, 3.4 mm) around the optic nerve with a minimum of 50 automatic real time repetitions, respecting OSCAR-IB criteria (Schippling et al., 2015), and a macular scan consisting of 25 vertical scans centered on the fovea. The relevant OCT parameters global pRNFL, total macular volume, and all intra-retinal layer volumes were obtained with the help of macular segmentation. Macular segmentation was performed with Spectralis Viewing Module V. 6.0.9.0 provided by Heidelberg Engineering. As described previously (Balk et al., 2014), the mean volume within the perifoveal rim was calculated for the pRNFL (macular retinal nerve fiber layer), mGCL (macular ganglion cells layer), mIPL (macular inner plexiform layer), mINL (macular inner nuclear layer), mOPL (macular outer plexiform layer), and mONL (macular outer nuclear layer). We calculated the ratio ONL central/mean (OPL nasal; OPL temporal) based on macular volume data (Albrecht et al., 2012). OCT was intended to be performed in patients but not in controls of the study.

#### Magnetic resonance imaging

In accordance with a prior study assessing midbrain atrophy in patients with PSP (Cosottini et al., 2007), we determined the ratio of the midbrain cross-sectional area scaled by the pons area (A_midbrain_/A_pons_). All measurements were performed using the Hermes Viewer (Hermes Medical Solutions AB, Stockholm, Sweden) on mid-sagittal images of T1w 3-dimensional sequences with an isotropic voxel resolution of 1 × 1 × 1 mm^3^. A mid-sagittal plane of the brain volume passing through the middle of the interpeduncular fossa, the middle of the aqueduct, and the falx cerebri was chosen (each determined on axial images). The midbrain area was determined by tracing the contour of the midbrain down to a line parallel to the hypothetic conjunction between the genu and splenium of the corpus callosum touching the superior part of the pons. For the determination of the pons midsagittal area, we used the region underneath, extending from the lower bound of the midbrain area down to a parallel line touching the inferior border of the pons.

### Statistics

Demographic and semiquantitative PET results in different target VOIs were compared between PSP patient and control groups using a two-tailed Student's *t*-test. Effect sizes for the discrimination between PSP and healthy controls were calculated as Cohen's *d*. Multimodal assessments by OCT and MRI were compared against published discrimination thresholds for PSP or neurodegeneration on a single patient level, and are reported descriptively in relation to reported standard deviations of findings in healthy controls. Correlation analyses between PET SUVR_CBL_ in target VOIs and clinical parameters (PSPRS, SEADL, disease duration), as well as inter-modality correlations were performed using either Pearson's coefficient of correlation (R), or Spearman rank correlation (R_s_) as deemed appropriate after prior testing for normal distribution of data by Kolmogorov-Smirnov-Test. The single PSP-PNFA case was evaluated descriptively.

## Results

### Demographic and clinical data

PSP-RS patients and healthy controls were matched for age and education, as detailed in Table 1 that also shows that cognitive performance tended to be lower in the PSP group by about one MMSE point (*p* = 0.052), but was still in the range that is not regarded indicative of dementia. In the PSP-RS group, a wide range of clinical severity (PSPRS range 19–44, SEADL range 50–90) and of disease duration (7–60 months) was present. The suspected PSP-PNFA case was a female aged 65 years with PSPRS of 10, SEADL of 90, and disease duration of 22 months.

**Table 1:** Demographics and Clinical Presentation.

	**PSP (*N* = 11)**	**Healthy controls (*N* = 9)**
Age (y)	68.4 ± 7.4	71.6 ± 6.6
Gender (m/f)	5/6	5/4
Education (y)	13.2 ± 3.2	12.9 ± 2.5
MMSE (median, range)	27, 25–29	28, 26–30
PSPRS (median, range)	30, 19–44	n/t
SEADL (median, range)	60, 50–90	n/t
Disease Duration (mo)	36 ± 19	n/a

*Y, years; m, male; f, female; n/a, non applicable; n/t, non tested; MMSE, mini-mental state examination; PSPRS, progressive supranuclear palsy rating scale; SEADL, Schwab and England Activities of Daily Living scale; mo, months*.

### [^18^F]-THK5351 Pet imaging

#### Tracer kinetics and time window

**Figure 1:**
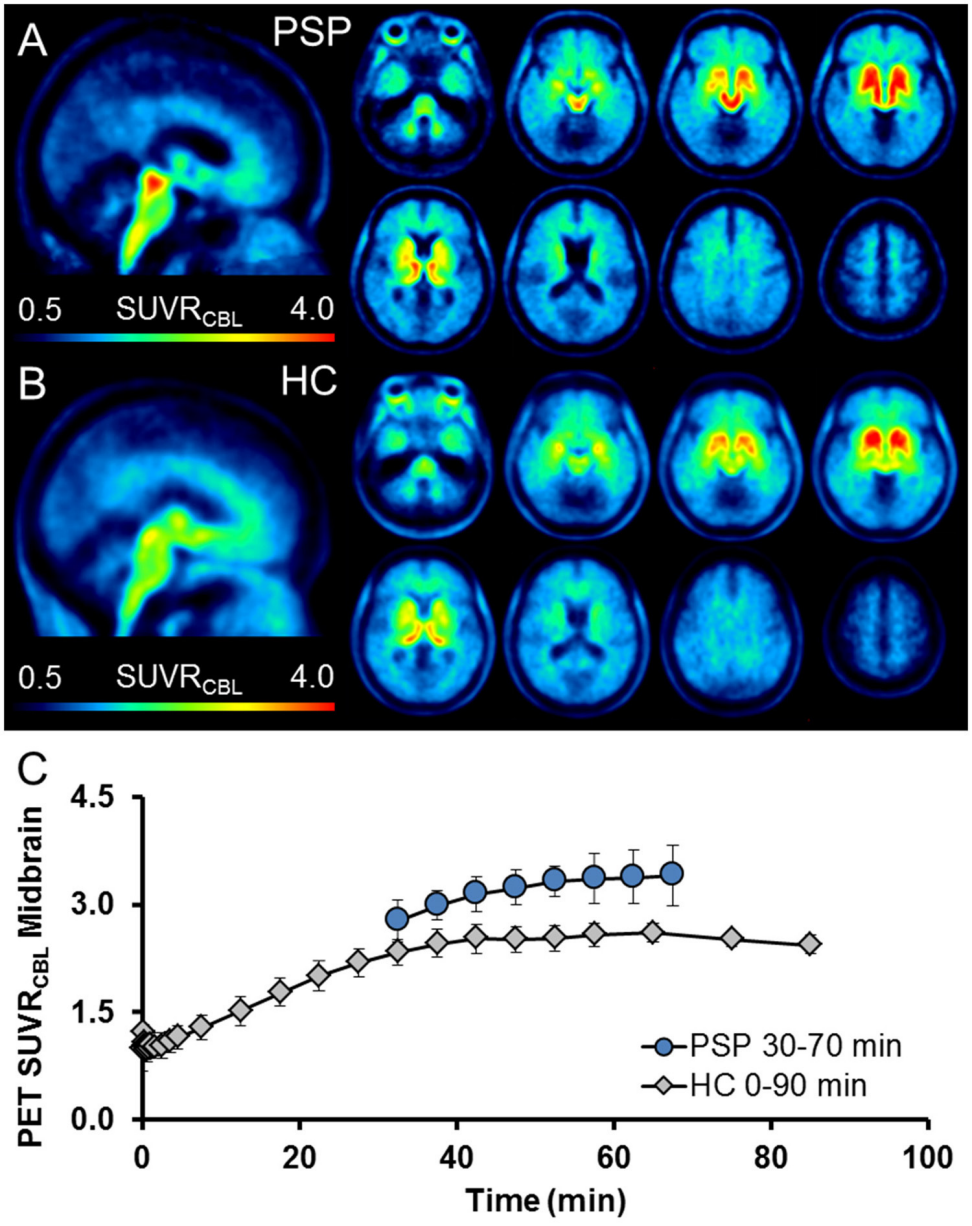
High midbrain uptake in sagittal slices allows visual discrimination of the PSP patient group **(A)** from the healthy control group (HC) **(B)**. Axial slices through the brain indicate high background binding in the basal ganglia and brainstem regions of HC, but further elevation in regions with known tau deposition in PSP vs. HC (midbrain, dentate nucleus, globus pallidus, frontal cortex). Images represent group averages. **(C)** Dynamic imaging indicates stable kinetics ≥ 50 min p.i. as illustrated by a persistently higher time-activity-curve (ratio), deriving from PSP patients in contrast to the mean for the healthy control group (mean ± SD). Data points represent an average of all counts in a defined time frame.

Higher [^18^F]-THK5351 retention was evident on visual inspection in the midbrain of the PSP-RS patient group when compared to controls (Figures 1A,B). Dynamic imaging in all subjects, controls as well as patients, confirmed highest stability in midbrain SUV for times exceeding 50 min p.i. (Figure 1C; slope increase: 3.1% for 50–70 min p.i. vs. 9.5% for 40–60 min p.i. window). We selected the 50–70 min post injection (p.i.) window for further static analyses.

#### Voxel-wise PET analyses

**Figure 2:**
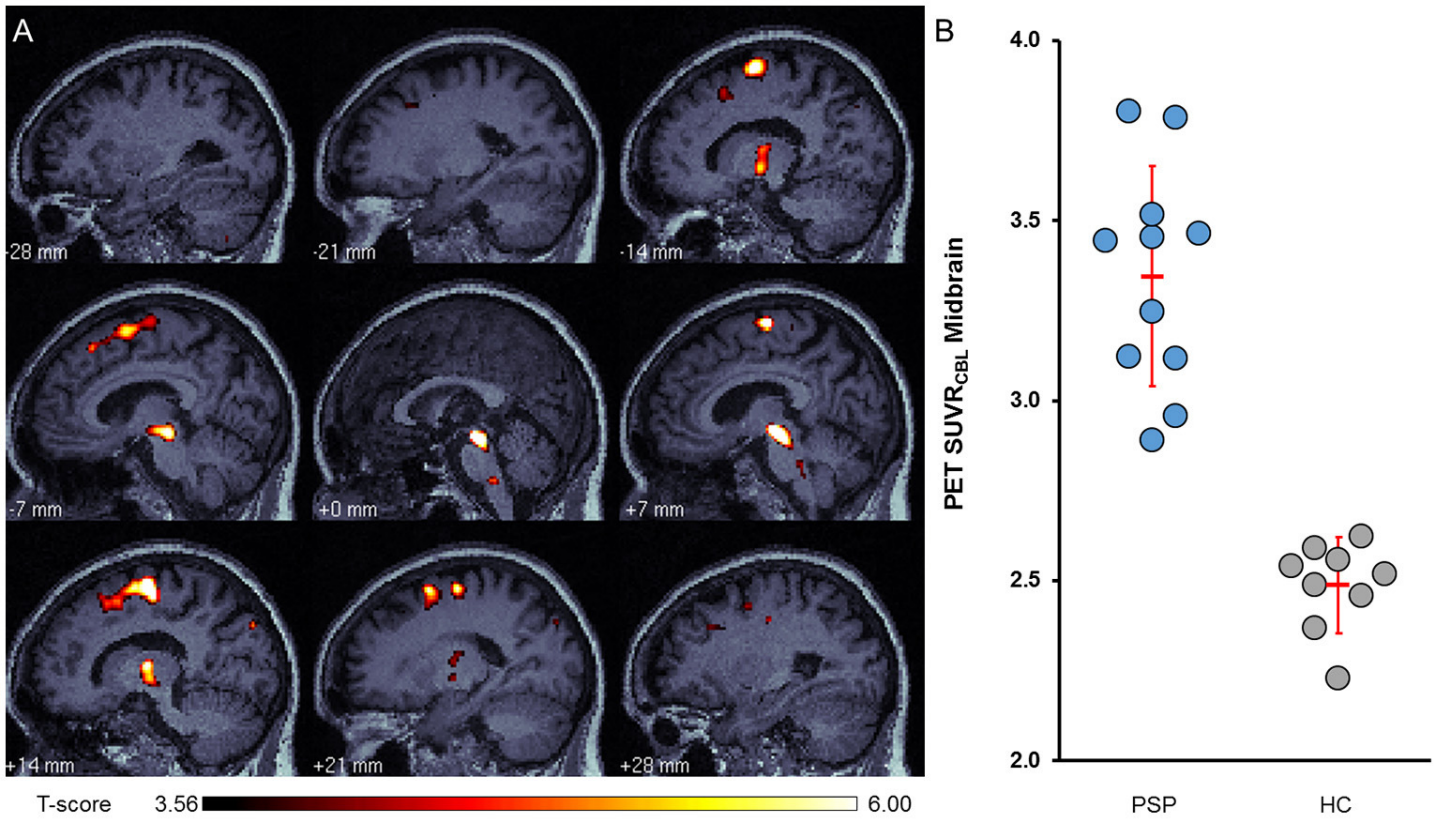
**(A)** Regional statistical parametric mapping of tau depositions in the whole cohort of PSP patients (*N* = 11) in contrast to healthy controls (HC; *N* = 9). Two-tailed *t*-test; significance threshold: *p* < 0.05 (FDR-corrected); *k* > 20 voxel. T-score maps are projected upon an in-house MRI template. **(B)** Quantitative single patient PET values from the resulting midbrain VOI indicating a high contrast without overlap between PSP and HC.

SPM analysis to investigate voxel-wise differences between clinical PSP-RS patient and healthy control groups showed significant clusters of elevated [^18^F]-THK5351 PET signal in the midbrain (412 voxels), bilateral globus pallidus (436 voxels), bilateral frontal cortex (2258 voxels), and the medulla oblongata (50 voxels) in patients vs. controls (Figure 2A; threshold: *p* < 0.05, FDR corrected).

#### VOI-based PET analyses

Semi-quantitative analysis indicated the strongest discrimination between PSP-RS and healthy controls for [^18^F]-THK5351 uptake in the midbrain (+35%; *p* = 3.01E-7; Cohen's *d* = 4.0; Figure 2B), followed by the frontal cortex (+28%; *p* = 1.73E-7; Cohen's *d* = 3.7), globus pallidus (+24%; *p* = 3.84E-7; Cohen's *d* = 3.6), and medulla oblongata (+20%; *p* = 1.01E-5; Cohen's *d* = 2.7) (Table 2, Supplementary Figure 1A). Results obtained using the predefined atlas VOIs mirrored the voxelwise results, albeit with lower effect size (Supplementary Table 1). The range of [^18^F]-THK5351 uptake values in the midbrain of patients did not overlap with control values, either for SPM defined or for predefined atlas VOIs.

**Table 2:** [^18^F]-THK5351 PET estimates in PSP-RS and HC.

**Brain region**	**SPM (voxels)**	**PSP-RS (SUVR_CBL_)**	**HC (SUVR_CBL_)**	**Difference (%)**	**Effect size (*d*)**
Midbrain	412	3.35 ± 0.31	2.49 ± 0.12	+35 ± 12	4.0
Frontal cortex	2258	1.51 ± 0.09	1.18 ± 0.09	+28 ± 7	3.7
Globus pallidus	436	3.02 ± 0.19	2.44 ± 0.14	+24 ± 8	3.6
Medulla oblongata	50	2.76 ± 0.16	2.30 ± 0.18	+20 ± 7	2.7

*SPM- and VOI-based results of [^18^F]-THK5351 PET in groups of PSP patients and HC. HC, Healthy controls; PSP-RS, progressive supranuclear palsy Richardson's Syndrome; SUVR, standardized-uptake-value-ratios; CBL, cerebellum; SPM, Statistical parametric mapping; VOI, volume-of-interest*.

**Figure 3:**
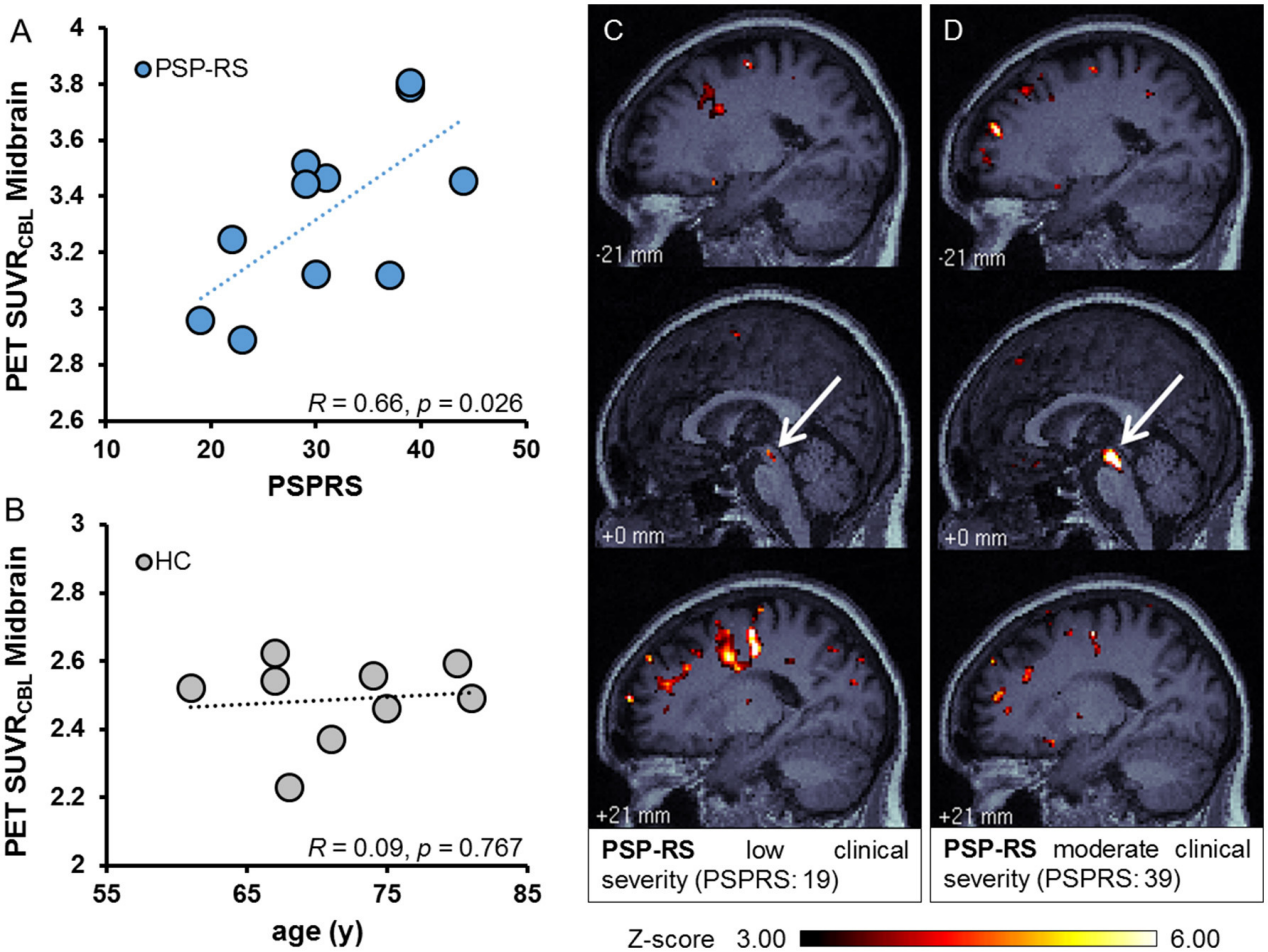
Positive correlation between quantitative [^18^F]-THK5351 PET results in midbrain with the clinical ratings by PSPRS **(A)**. Age was not correlated with tau-PET uptake in target regions as tested in healthy controls (HC), exemplified for the midbrain **(B)**. Examples of individual Z-score maps in a PSP-RS patient with low clinical severity (**C**; PSPRS 19) and only minor uptake in midbrain (highlighted by white arrow) in contrast to a PSP-RS patient with comparatively higher clinical severity (**D**; PSPRS 39) and strong tau accumulation in midbrain (highlighted by white arrow).

We tested the correlation of clinical severity scores with the [^18^F]-THK5351 PET SUVR from the four target regions in order to explore their possible associations with estimated regional quantification *in vivo*. Figure 3A illustrates the significant positive correlation between clinical severity (measured with PSPRS) and midbrain [^18^F]-THK5351 uptake (*R* = 0.66, *p* = 0.026), which was the brain region with the largest effect size with respect to tracer uptake. [^18^F]-THK5351 PET binding in the frontal cortex, globus pallidus, or medulla oblongata did not significantly correlate with PSPRS scores (see Supplementary Figure 1B). Significant correlations or trends were not found between SEADL scores or disease duration and [^18^F]-THK5351 PET signal in any brain region. Importantly, no correlations or trends between age and [^18^F]-THK5351 binding in target regions were observed in controls (exemplary midbrain: *R* = 0.09, *p* = 0.767; Figure 3B).

Individual Z-score maps indicated the ability of capturing the alterations of [^18^F]-THK5351 PET signal vs. healthy controls on the single patient level. Here, the correlation of clinical severity with midbrain binding was further substantiated, which is exemplified in individual cases with low and high PSPRS scores, respectively (compare Figures 3C,D). Notably binding in the frontal cortex did not follow the clinical severity and was sometimes even higher in cases with a low PSPRS when compared to cases with high PSPRS (compare Figures 3C,D). This was also reflected by the correlation between PSPRS and frontal cortical [^18^F]-THK5351 PET signal (*R* = −0.13, *p* = 0.707).

**Figure 4:**
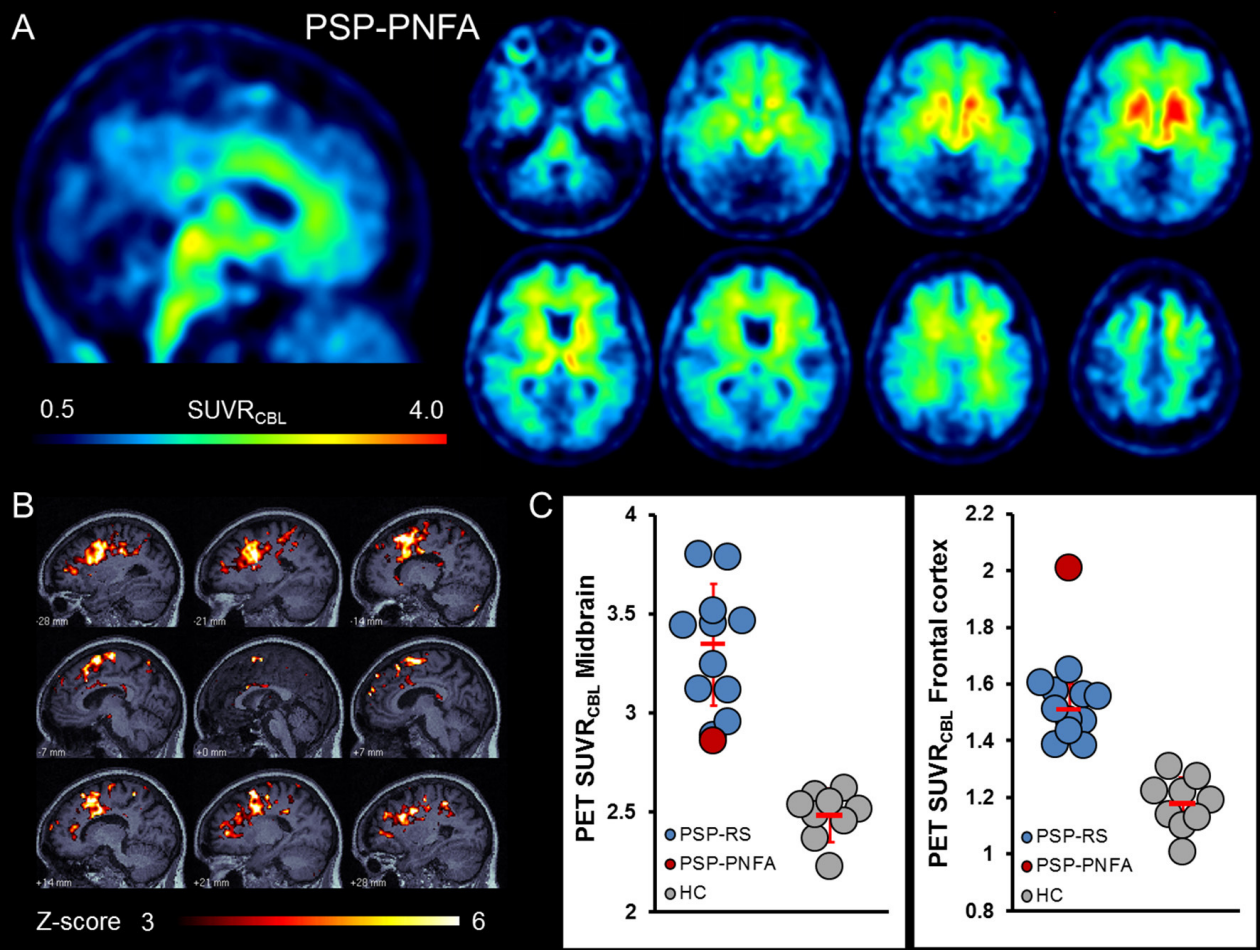
Sagittal and axial slices are presented in analogy to Figure 1, and therefore afford a visual comparison of PSP-PNFA, PSP-RS, and HC **(A)**. The most impressive finding was the strong uptake in the frontal cortex in PSP-PNFA, which was as well very prominent in voxel-wise individual Z-score analysis vs. the HC group **(B)**. Quantitative VOI-based analysis indicated a midbrain signal at the bottom of PSP-RS cases, but the (by far) highest [^18^F]-THK5351 PET signal in the frontal cortex frontal of all studied cases **(C)**. Clinical severity was low at the time of the PET scan (PSPRS: 10), but had substantially increased at 1 year follow-up (PSPRS: 38).

The single case of suspected PSP-PNFA (PSPRS 10 at time of PET scan) had a rather different distribution pattern, which was characterized by accumulation of the tracer predominantly in the frontal cortex and only minor subcortical binding (Figures 4A,B). Quantitatively, the (by far) highest [^18^F]-THK5351 PET signal was in cortex (SUVR 2.01 vs. 1.51 ± 0.09 in the remaining patients), with little evidence of elevated midbrain [^18^F]-THK5351 accumulation (Figure 4C; SUVR 2.86 vs. 3.35 ± 0.31 in remaining patients vs. 2.49 ± 0.12 in healthy controls). Clinical follow-up 1 year after the PET scan indicated a dramatic disease progression (PSPRS: 38), and resulted in a definitive diagnosis of PSP-PNFA.

### Correlation with other techniques: optical coherence tomography and magnetic resonance imaging

For multimodal comparison of [^18^F]-THK5351 PET and for more detailed classification of the patient group, we undertook assessment by OCT (in 8/11 PSP-RS patients) and MRI (9/11) in comparison with findings in normal subjects in the literature. OCT was not feasible in three patients due to impaired fixation. Two patients refused MRI.

**Figure 5:**
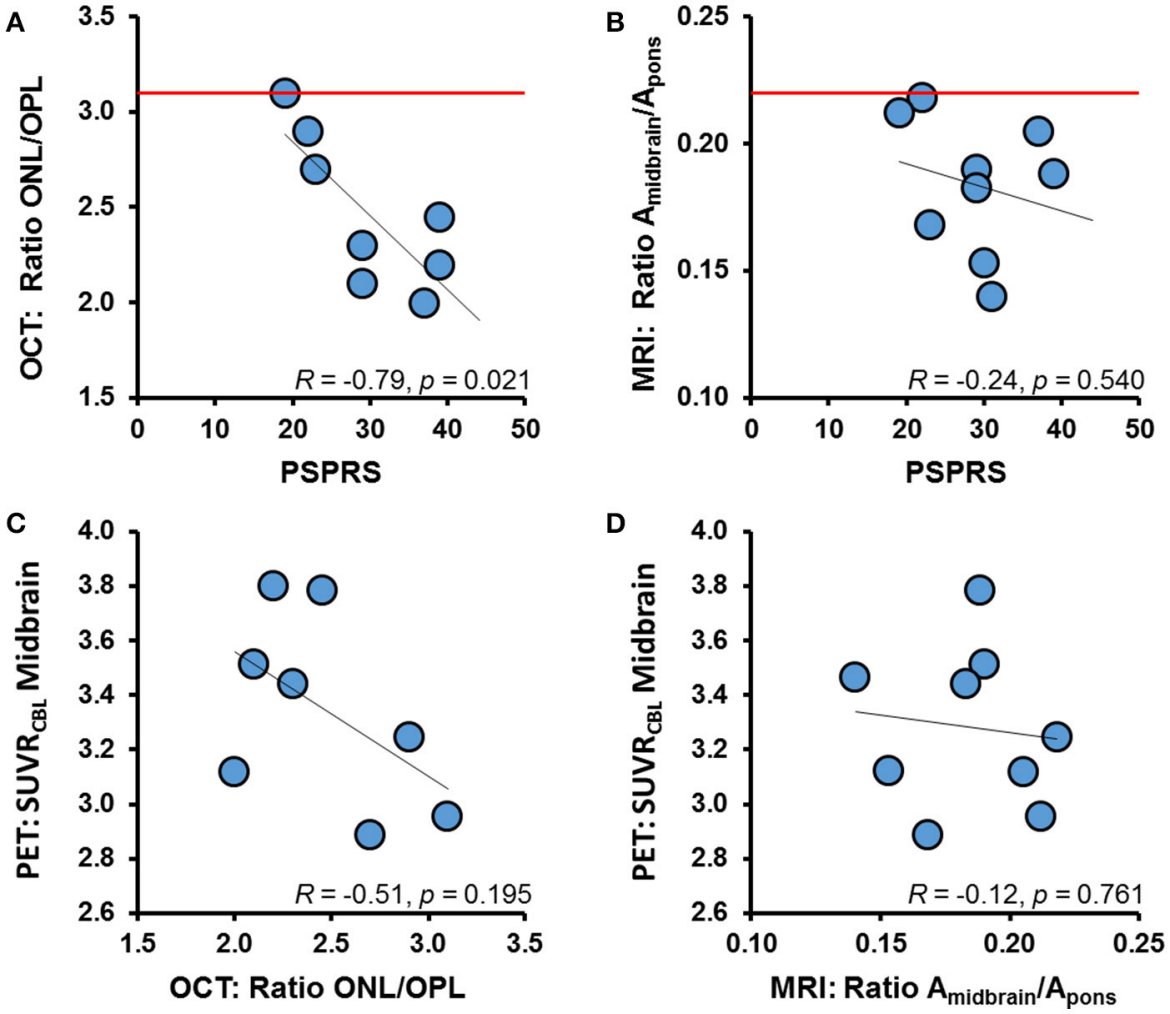
Resulting values of OCT **(A)** and MRI **(B)** are illustrated for each examined PSP patient together with the published discrimination threshold against healthy controls (red line). Linear correlations between clinical severity (PSPRS) and OCT/MRI are indicated by black lines. Inter-modality correlations are shown for OCT and [^18^F]-THK5351 PET **(C)** as well as for MRI and [^18^F]-THK5351 PET **(D)** and indicated by black lines.

OCT revealed ONL/OPL ratios in a range of 2.2 SD below the published discrimination threshold (Albrecht et al., 2012) (vs. Parkinson's disease data) for confirmation of PSP in all eight patients. Seven of eight patients ranged clearly below the threshold and one at borderline value (Figure 5A). ONL/OPL values correlated inversely with clinical severity by PSPRS (*R* = 0.79; *p* = 0.024). The midbrain-to-pons ratio calculated from MRI was decreased by as much as 2 SD (Cosottini et al., 2007) in all PSP patients, although there was no significant correlation between MRI findings and clinical symptom severity (Figure 5B).

ONL/OPL ratios indicated a trend toward negative correlation with the [^18^F]-THK5351 PET signal in the midbrain (*R* = 0.51; *p* = 0.195; Figure 5C), whereas the midbrain-to-pons ratio deriving from MRI did not show a clear association with [^18^F]-THK5351 PET binding (*R* = −0.12; *p* = 0.761; Figure 5D).

## Discussion

We present the first dedicated [^18^F]-THK5351 PET study in a clinically well-characterized group of PSP-RS patients and demonstrate that enhanced *in vivo* tracer uptake in the midbrain clearly discriminates PSP-RS patients from age-matched HCs. We also found, on voxel-wise analysis, that in living patients there are patterns of tracer uptake distribution that parallel the *post mortem* findings of lesions in PSP-RS. This suggests the suitability of the method for diagnostic and maybe for disease monitoring purposes. Furthermore, our data indicate a correlation between midbrain [^18^F]-THK5351 PET signal and clinical severity.

In the entire group of patients with a clinical diagnosis of suspected PSP, the mean [^18^F]-THK5351 PET signal was significantly elevated in midbrain, bilateral globus pallidus, bilateral frontal cortex, and medulla oblongata. This distribution pattern is an excellent match to the topology of tau deposition known from histological examination *post mortem* (Williams et al., 2007). The [^18^F]-THK5351 PET signal in the dentate nucleus as another tau accumulating region in PSP was also elevated visually but did not survive the significance threshold for multiple comparisons, probably due to the relatively lower pathology in this region and due to the small size suffering from larger partial volume effects. Nonetheless, possible contamination of the cerebellar cortex reference region by spill-over is a potential confound in individuals with high tau deposition in the dentate nucleus, as is typical in cerebellar variants of PSP (Kanazawa et al., 2009). The range of clinical severity in the patients (PSPRS 31 ± 8, range 19–44), is a strength of the present study, as it represents a clinically less affected group than the PSP patients of a previous report with the alternative PET tracer [^18^F]-FDDNP (PSPRS 42.4 ± 9.5) (Kepe et al., 2013). Thus, [^18^F]-THK5351 discriminates PSP patients from controls even at earlier disease-stages, yet the exact detection threshold can only be defined in a larger cohort. Interestingly, we obtained an initial impression of the fitness of this tracer for molecular imaging of non-RS PSP; our single case of the rare disease entity PSP-PNFA showed slightly elevated midbrain tracer accumulation, and very high [^18^F]-THK5351 PET signal in cortex (Figure 4). This finding corresponds well with the clinical course of this rare disease subtype (5% of PSP-tau), manifesting with non-fluent aphasia some years before the onset of typical PSP motor symptoms. Our findings are further supported by the documented gray matter atrophy in the same prefrontal regions to volumetric MRI examination of four PSP-PNFA cases (Rohrer et al., 2010). Although we cannot draw strong conclusions from a single case, we have preliminary evidence that the heterogeneity of PSP variants can be depicted by [^18^F]-THK5351 PET. The main findings of the study are encouraging for the use of [^18^F]-THK5351 PET for PSP diagnosis in atypical or clinically ambiguous cases.

We found a significant correlation between the intensity of the [^18^F]-THK5351 midbrain signal and disease severity, as estimated by PSPRS (Figure 3A). This finding emphasizes the clinical potential of this biomarker through imaging of the symptom-related disease related pathology in individual patients. It reflects on the predominance of core symptoms of PSP-RS for disease severity caused by local tau pathology in the midbrain (Golbe and Ohman-Strickland, 2007). Absent correlations for other brain regions could be due to insufficient statistical power, but might also reflect region-specific associations with distinct symptoms, perhaps better investigated in a larger group of patients. Importantly, very recent findings of MAO-B interaction of [^18^F]-THK5351 have to be taken into account when interpreting the current results (Ng et al., 2017). We note that further studies need to address which fraction of [^18^F]-THK5351 binding in the brain of PSP patients is caused by specific tau deposit binding and which fraction is related to off-target MAO-B binding, which might be as well-elevated due to concomitant neuroinflammation (Zimmer et al., 2014). Nonetheless the correlation of tracer signal with clinical severity at least assures us about well-capturing the disease stage. Investigations of [^18^F]-THK5351 PET as a disease biomarker must accommodate possible normal age-dependent changes in healthy brain. We found no correlation between [^18^F]-THK5351 midbrain binding and age in our group of healthy controls (Figure 3B). This is in agreement with an earlier finding for the tau binding component of [^18^F]-AV1451 (Schöll et al., 2016). The slightly greater age of our control group supports the unambiguous attribution of group differences to tau pathology in the PSP group. A preliminary comparison of tau-PET with other modalities is provided in the Supplementary Material.

Off-target binding as already mentioned above may contribute to the uptake patterns of newly developed tau-ligands. In particular, the pyridoindole ligand [^18^F]-AV1451 and the arylquinoline ligand [^18^F]-THK5351 both have unexpectedly high SUVR in the basal ganglia and brainstem structures of healthy volunteers (Betthauser et al., 2016), which is likely related to off-target binding (Marquié et al., 2015). In our recent analysis, we found that off-target binding to neuromelanin or MAO-B can explain [^18^F]-THK5351 off-target retention in the midbrain. We are unaware of any grounds to expect important differences in neuromelanin binding in the substantia nigra of Caucasians as opposed to Asians. Furthermore, in PSP, neuromelanin- containing neurons of the substantia nigra are undergoing degeneration as part of the natural course of the disease. As such, this non-specific binding component would be expected to decrease in PSP. That we observe the opposite is evidence that we are detecting a real increase caused by tau pathology. Interestingly, reduced off-target binding of [^18^F]-AV1451 in the substantia nigra reveals the degeneration of pigmented dopamine neurons in Parkinson's disease patients (Hansen et al., 2016). Thus, it is crucial to establish the regional specificity of tau-tracers for tau deposition in PSP and other neurodegenerative diseases. Another off-target of [^18^F]-THK5351 is considered to be MAO-B (Ng et al., 2017). In the autoradiography of healthy control brain, we observed [^18^F]-THK5351 off-target binding in the basal ganglia and MAO-B inhibitors can completely block these [^18^F]-THK5351 binding (manuscript in preparation). As a neuroinflammatory component of taupathies is well-known we acknowledge that our elevated PET signal in PSP patients may be a composite of tau and MAO-B binding. Compared to [^18^F]-AV1451, no off-target [^18^F]-THK5351 binding exists in the choroid plexus.

In a recent [^18^F]-AV1451 study the cerebral signal was higher in PSP patients compared to healthy controls, which matched the known disease topology and clinical severity (Whitwell et al., 2016), as in the present [^18^F]-THK5351 study. However, two other recent [^18^F]-AV1451 studies revealed significantly elevated SUVR in the basal ganglia of PSP patients when compared to healthy controls, without clear relation to the expected disease pathology, and not correlating with clinical severity (Cho et al., 2016; Smith et al., 2016a). Notably, PET examination with this tracer clearly distinguishes PSP from Parkinson's disease and Alzheimer's disease patients (Cho et al., 2016; Whitwell et al., 2016). However, autoradiography in one of these studies did not confirm the specific binding of [^18^F]-AV1451 to tau aggregates. Another *in vitro* study indicated low displaceable binding to PSP tau in brain sections, with no correlation between tracer binding *in vitro* and quantitative tau load (Sander et al., 2016). On the other hand, [^18^F]-THK5351 had specific binding to PSP tissue examined by autoradiography *post mortem*, which matched tau deposition to immunohistochemistry (Ishiki et al., 2016). Present data are in line with the *in vitro* findings, and generally support the fitness of [^18^F]-THK5351 PET for the sensitive detection of tau deposits, with the caveat that high background in controls most likely reflects an off-target binding component.

In line with the present results with the arylquinoline ligand [^18^F]-THK5351, others have reported elevated PET signal with this tracer in a single PSP case (Chiotis et al., 2016). Another report did not observe specific autoradiography binding in tissue of a single PSP patient for both tritiated tracers (Smith et al., 2016b), which might be related to methodological factors *in vitro*. Specific binding to 4R tau *in vitro* would be influenced by the particular tracer concentration and washing procedures, for example. Earlier findings in patients with corticobasal syndrome are discussed in the Supplementary Material.

Among the limitations of the current investigation we note the lack of histopathological validation which reflects the moderate clinical severity within our patients, all of whom are alive at the time of writing. However, the present results have face validity given the high agreement between [^18^F]-THK5351 PET findings and known topology of tau distribution. We further note that data from PSP patients and healthy controls were derived from centers using different PET scanners, albeit of similar performance. Confounds due to minor differences in PET resolution and partial volume effects, as well as effects deriving from differing subject ethnicity, cannot be fully excluded. We acknowledge also the small sample size of PSP patients imaged by [^18^F]-THK5351 PET. Amyloid status was not assessed for patients (8/9 of HC had an amyloid scan available which was negative in all cases), which limits the interpretation of [^18^F]-THK5351 PET in the unlikely event of coexisting Alzheimer's disease pathology, although this is not expected due to excellent specificity of the NINDS-SPSP diagnostic criteria for the diagnosis of PSP (Respondek et al., 2014). Despite these considerations, we find the method proves robust for the intended discrimination, and suggest that the present results justify to initiate a larger multicenter investigation of this rare disease.

## Author contributions

MB and SS: Research project: organization and execution; Statistical analysis: execution; Manuscript preparation: writing of the first draft. GH, AL, LG, Statistical analysis: review and critique; Manuscript preparation: review and critique. SL, JH, JB, GR, and GN: Research project: execution; Manuscript preparation: review and critique. JS: Statistical analysis: execution; Manuscript preparation: review and critique. CZ: Statistical analysis: design; Manuscript preparation: review and critique. FV: Statistical analysis: execution; Manuscript preparation: review and critique. PB: Statistical analysis: review and critique; Manuscript preparation: review and critique. KF, AI, KB, and AD: Research project: organization; Manuscript preparation: review and critique. NO: Research project: organization; Statistical analysis: review and critique; and Manuscript preparation: review and critique. JL and AR: Research project: conception and organization; Statistical analysis: design, review and critique; and Manuscript preparation: review and critique.

### Conflict of interest statement

A. E. Lang has served as an advisor for Abbvie, Acorda, Avanir Pharmaceuticals, Bristol Myers Squibb, Cipla, Intekrin, and Merck; received honoraria from Sun Pharma, Medichem, Medtronic, Teva, UCB, AbbVie; received grants from Brain Canada, Canadian Institutes of Health Research, Edmond J Safra Philanthropic Foundation, Michael J. Fox Foundation, the Ontario Brain Institute, National Parkinson Foundation, Parkinson Society Canada, and W. Garfield Weston Foundation; received publishing royalties from Saunders, Wiley-Blackwell, Johns Hopkins Press, and Cambridge University Press. A. Golbe: received research support from Bristol-Myers Squibb, AbbVie, American Parkinson's Disease Association, Movement Disorders Research Fund of Rutgers University. Serves as a consultant for Bristol-Myers Squibb, AbbVie. P. Bartenstein received speaking honoraria from Simens and GE Healthcare. N. Okamura is a consultant for Clino Corp., receives royalties from GE Healthcare Corp., and is funded by Grant-in-Aid for Scientific Research (B) (15H04900) and Scientific Research on Innovative Areas (26117003) of the Ministry of Education, Culture, Sports, Science and Technology (MEXT), Japan. J. Levin received research support from DZNE, speaker's fees from Bayer and serves as a consultant for Aesku and Hexal. A. Rominger: received speaking honoraria from GE Healthcare and Piramal Imaging. The other authors declare that the research was conducted in the absence of any commercial or financial relationships that could be construed as a potential conflict of interest.

## Abbreviations

HC: Healthy controls
MAPT: microtubule-associated protein tau
MMSE: Mini-Mental State Examination
MNI: Montreal Neurological Institute
MRI: magnetic resonance imaging
OCT: optical coherence tomography
ONL/OPL: outer nuclear layer/outer plexiform layer
PET: positron emission tomography
PSP: progressive supranuclear palsy
PSPRS: PSP rating scale
PSP-RS: PSP-Richardson's syndrome
PSP-PNFA: PSP-progressive non-fluent aphasia
RNFL: retinal nerve fiber layer
SC: solvent containers
SEADL: Schwab and England Activities of Daily Living scale
SPM: statistical parametric mapping
SUVR: standard-uptake-value ratio
VOI: volume-of-interest.
