# Supplementary material for: [18F]-THK5351 PET Correlates with Topology and Symptom Severity in Progressive Supranuclear Palsy
Source: Front Aging Neurosci. 2018 Jan 17;9:440. doi: 10.3389/fnagi.2017.00440 (PMC5776329; doi:10.3389/fnagi.2017.00440)
Supplement: Supplementary file 1 [file DataSheet1.docx]

**Supplementary Material**

**Supplemental Methods**

Detailed description of the radiosynthesis: Automated production of [^18^F]-THK5351 was performed on a Raytest^®^ SynChrom R&D single reactor synthesizer. Solvent containers were loaded with reagents, and cartridges were assembled on the synthesizer. The manufacturing process was performed automatically using the Raytest^®^ control software. No-carrier-added ^18^F-fluoride was produced via ^18^O(p, n)^18^F reaction by proton irradiation of ^18^O-enriched water, and was directly delivered to an ion exchange cartridge (Chromabond PS-HCO_3_^-^, Macherey Nagel, Dueren, Germany). The trapped ^18^F-fluoride was eluted into the reactor using a mixture of Kryptofix^®^ 222 (12.5 mg), potassium carbonate (12.5 µL, 1 M), water (187.5 µL) and acetonitrile (800 µL). The solution was evaporated to dryness by azeotropic distillation, and the drying process was repeated after addition of acetonitrile (0.8 mL). The precursor (2 mg) was transferred in dimethylsulfoxide (0.7 mL) to the reactor, and the mixture was heated at 110°C for 10 minutes. HCl (0.2 mL, 2 M) was added, and the mixture stirred for 3 min. at 110°C. After quenching with AcOK (1 mL, 4 M) in H_2_O (4 mL), the mixture was transferred to a SepPak tC18 Plus Short cartridge (Waters GmbH, Eschborn, Germany), which was then washed with H_2_O (5 mL). Radioactive products were eluted with EtOH/H_2_O 1:1 (4 mL) and purified via semi-preparative HPLC (Inertsil ODS-4 C18 column, 250 x 10 mm, 5 µm; isocratic elution with 75% NaH_2_PO_4_ (20 mM) / 25% acetonitrile; flow: 5 ml/min; UV-detection: 360 nm). The HPLC purified product peak was collected, diluted with H_2_O (20 mL) and ascorbic acid (0.5 mL, 25%) and passed through a tC18 SepPak Plus Short cartridge (Waters GmbH, Eschborn, Germany). The radiolabelled product was washed with H_2_O (4 mL), eluted with ethanol (1 mL) into the product vial, diluted with PBS (9 ml) and filtered through a sterile filter (Cathivex GV^®^, 0.2 µm, Merck Millipore). Purity was confirmed via analytical HPLC (Inertsil ODS-4 C18 column, 150 x 4.6 mm, 5 µm; isocratic elution with 65% NH_4_OAc (10 mM) / 35% acetonitrile; flow: 1.5 ml/min; UV-detection: 360 nm). The radiochemical yield was 12±4% (not decay-corrected, n=10) and radiochemical purity >99% at the end of the 84 min synthesis.

**Supplemental Figure 1:** (**A**) Plots show quantitative single patient PET values from the resulting VOIs in the frontal cortex, globus pallidus, and medulla oblongata indicating a high contrast between PSP and HC. (**B**) Correlation between quantitative PET results in the frontal cortex, globus pallidus, and medulla oblongata did not indicate a significant association with PSPRS.


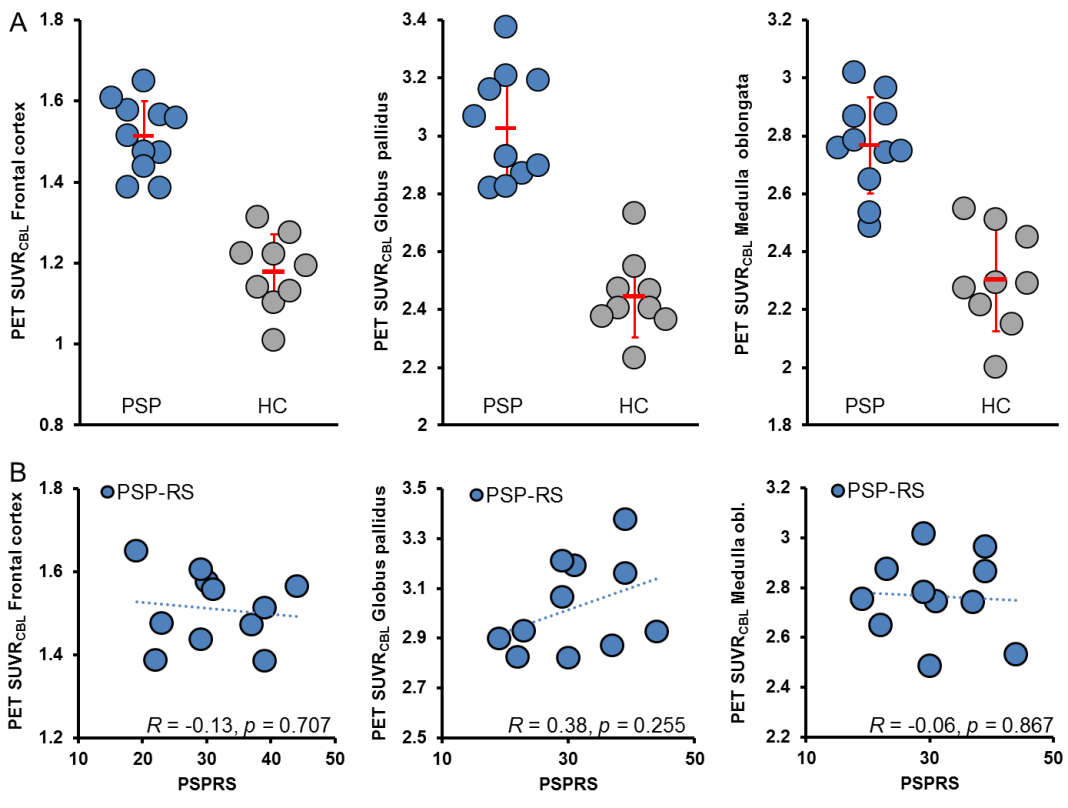


**Supplemental Table 1:** Predefined volume-of-interest (VOI) based results of [^18^F]-THK5351 PET in groups of PSP patients and healthy controls (HC) as obtained from VOIs of the Hammers atlas ([Hammers et al., 2003](#_ENREF_14)). Regions were selected in analogy to the SPM-defined VOIs. The midbrain VOI was created by masking voxels of the brainstem atlas VOI below the upper edge of the pons. SUVR: standardized-uptake-value-ratios; CBL: cerebellum. L = left; R =right; *p < 0.05; ** p < 0.01.

| **Region (Hammers Atlas)** | **PSP**  **(SUVR_CBL_)** | **HC**  **(SUVR_CBL_)** | **Difference**  **(%)** | **Effect size**  **(d)** | **p-value** |
| --- | --- | --- | --- | --- | --- |
| Precentral gyrus L | 1.24 ± 0.07 | 1.18 ± 0.10 | 5.7% | 0.83 | 0.0805 |
| Precentral gyrus R | 1.26 ± 0.05 | 1.15 ± 0.10 | 10.0% | 1.62 | 0.0022** |
| Inferior frontal gyrus L | 1.23 ± 0.06 | 1.25 ± 0.08 | -2.3% | -0.42 | 0.3569 |
| Inferior frontal gyrus R | 1.27 ± 0.07 | 1.20 ± 0.10 | 6.2% | 0.87 | 0.0677 |
| Superior frontal gyrus L | 1.28 ± 0.05 | 1.21 ± 0.11 | 5.6% | 0.81 | 0.0977 |
| Superior frontal gyrus R | 1.29 ± 0.06 | 1.21 ± 0.10 | 6.5% | 1.00 | 0.0390* |
| Putamen L | 2.58 ± 0.18 | 2.57 ± 0.26 | 0.2% | 0.02 | 0.9662 |
| Putamen R | 2.58 ± 0.20 | 2.49 ± 0.26 | 3.6% | 0.39 | 0.3978 |
| Globus pallidus L | 3.42 ± 0.30 | 3.09 ± 0.33 | 10.9% | 1.06 | 0.0288* |
| Globus pallidus R | 3.52 ± 0.33 | 3.20 ± 0.30 | 9.9% | 0.99 | 0.0418* |
| Substantia nigra L | 3.07 ± 0.46 | 2.62 ± 0.14 | 17.5% | 1.52 | 0.0102* |
| Substantia nigra R | 2.99 ± 0.39 | 2.47 ± 0.19 | 21.0% | 1.77 | 0.0020** |
| Brainstem | 2.13 ± 0.13 | 1.97 ± 0.11 | 8.0% | 1.34 | 0.0086** |
| Midbrain (from brainstem) | 2.68 ± 0.18 | 2.30 ± 0.12 | 16.7% | 2.62 | 2.69E-5** |

**Supplemental Discussion**

*Comparison of tau-PET with other modalities*

Clinical, neuropsychological, and volumetric MRI measurements are sufficiently sensitive to track disease progression of PSP upon annual follow-up, and constitute the current state of the art to monitor disease progression in studies aiming at disease-modifying therapies (Bang et al., 2016). The potential of [^18^F]-THK5351 PET to supersede the existing biomarkers cannot be confirmed in the present study with only eleven patients. Nonetheless, as tau aggregation is purported to be central to the pathogenesis of PSP, we expect that [^18^F]-THK5351 PET imaging presents valuable addition to current biomarkers. In support of multimodal comparisons, we had carried out additional optical coherence tomography (OCT) and structural MRI analyses in subsets of patients. OCT is an inexpensive, rapid, and non-invasive method to assess retinal morphology and thinning. Preliminary OCT data in neurodegenerative disorders had suggested that the inner nuclear layer (INL) and outer nuclear layer (ONL) of the retinal macula may be of special interest in parkinsonian syndromes, as they showed opposite changes (in thickness) for Parkinson’s disease and PSP patients (Albrecht et al., 2012). Indeed, all eight PSP examined patients showed an ONL/OPL ratio falling at or below the proposed cut-off (max. ~2.2 SD) (Albrecht et al., 2012). Additionally, the OCT results correlated well with clinical severity in PSP-RS patients and indicated a trend towards correlation with the [^18^F]-THK5351 PET signal, which further supports its use in disease monitoring. However, we note that our OCT measurements may have lower sensitivity as they were based on segmentation data of macular volume scans instead of single horizontal macular scans, as in a previous report (Albrecht et al., 2012). Furthermore, we note that OCT alone is unlikely to have specificity to discriminate among all parkinsonian syndromes. Thinning of retinal layers is more likely to reflect on neurodegenerative processes in general, rather than specific disease entities. Regarding our volumetric MRI analysis, midbrain volumes or ratios did not significantly correlate with the midbrain [^18^F]-THK5351 signal or clinical severity, which further supports the particular value of PET in the field of PSP neuroimaging.

*Earlier PET findings in patients with corticobasal syndrome*

Despite uncertainties related to the specific binding to PSP tissue *in vitro*, a PET investigation with [^18^F]-AV1451 in corticobasal syndrome reported a high correlation between the SUVR with tau burden measured *post mortem* (Josephs et al., 2016), as likewise shown for [^18^F]-THK5351 in corticobasal syndrome (Kikuchi et al., 2016). Thus, both ligands gave congruent results for corticobasal syndrome, whereas the topographic pattern of the two ligands did not match entirely in the contrast between PSP patients and healthy controls. Resolving this discrepancy might require head-to-head dual tracer studies, ideally in conjunction with subsequent autopsy validation and/or pharmacological blockade of MAO-B. Additionally, the carbon-11 labelled tau ligand [^11^C]-PBB_3_ has shown promising results in the discrimination of PSP cases from healthy controls, and revealed the tau distribution patterns expected from autopsy studies (Perez Soriano et al., 2016; Shinotoh et al.). However, by avoiding the requirement for an on-site cyclotron, fluorine-18 tracers will likely continue to play the major role in clinical routine imaging.

**Supplemental References**

Albrecht, P., Muller, A.K., Sudmeyer, M., Ferrea, S., Ringelstein, M., Cohn, E., Aktas, O., Dietlein, T., Lappas, A., Foerster, A., Hartung, H.P., Schnitzler, A., Methner, A., 2012. Optical coherence tomography in parkinsonian syndromes. PLoS One 7, e34891.

Bang, J., Lobach, I.V., Lang, A.E., Grossman, M., Knopman, D.S., Miller, B.L., Schneider, L.S., Doody, R.S., Lees, A., Gold, M., Morimoto, B.H., Boxer, A.L., Investigators, A.L., 2016. Predicting disease progression in progressive supranuclear palsy in multicenter clinical trials. Parkinsonism Relat Disord.

Josephs, K.A., Whitwell, J.L., Tacik, P., Duffy, J.R., Senjem, M.L., Tosakulwong, N., Jack, C.R., Lowe, V., Dickson, D.W., Murray, M.E., 2016. [18F]AV-1451 tau-PET uptake does correlate with quantitatively measured 4R-tau burden in autopsy-confirmed corticobasal degeneration. Acta Neuropathol 132, 931-933.

Kikuchi, A., Okamura, N., Hasegawa, T., Harada, R., Watanuki, S., Funaki, Y., Hiraoka, K., Baba, T., Sugeno, N., Oshima, R., Yoshida, S., Kobayashi, J., Ezura, M., Kobayashi, M., Tano, O., Mugikura, S., Iwata, R., Ishiki, A., Furukawa, K., Arai, H., Furumoto, S., Tashiro, M., Yanai, K., Kudo, Y., Takeda, A., Aoki, M., 2016. In vivo visualization of tau deposits in corticobasal syndrome by 18F-THK5351 PET. Neurology 87, 2309-2316.

Perez Soriano, A., Arena, J., Smith-Forrester, J., Vafai, N., Shahinfard, E., Miao, Q., Schaffer, P., Shinotoh, H., Higuchi, M., Sossi, V., Stoessl, A., 2016. Tau Imaging in Atypical Parkinsonism: Preliminary Evidence with [11C]PBB3 PET in PSP Subjects (S5.001). Neurology 86.

Shinotoh, H., Shimada, H., Hirano, S., Furukawa, S., Eguchi, Y., Takahata, K., Kimura, Y., Takano, H., Yamada, M., Kuwabara, S., Ito, H., Suhara, T., Higuchi, M., Imaging of tau pathology in patients with non-Alzheimer's disease tauopathies by [11C]PBB3-PET. Alzheimer's & Dementia: The Journal of the Alzheimer's Association 10, P6-P7.
